# Analysis of bacterial communities associated with potting media

**DOI:** 10.1186/s40064-016-1729-0

**Published:** 2016-01-26

**Authors:** A. M. Al-Sadi, H. A. Al-Zakwani, A. Nasehi, S. S. Al-Mazroui, I. H. Al-Mahmooli

**Affiliations:** Department of Crop Sciences, College of Agricultural and Marine Sciences, Sultan Qaboos University, PO Box 34, 123 Al Khod, Oman

**Keywords:** Contamination, Phylogeny, Potting media, 16S rRNA

## Abstract

**Background:**

Potting media are commonly used by growers in different parts of the world for potted plants, raising seedlings and for improving soil characteristics. This study was conducted to characterize bacterial communities occurring in 13 commercial potting media products originating from seven countries.

**Findings:**

Bacteria were isolated using serial dilution. Identification to the species level was based on phylogenetic analysis of the 16S rRNA gene. The analysis showed the association of 13 bacterial species with the different potting media samples, namely *Arthrobacter livingstonensis*, *Kocuriaflava*, *Leifsonia lichenia*, *Bacillus vallismortis*, *Bacillus pumilus*, *Staphylococcus warneri*, *Burkholderia phenazinium*, *Burkholderia* sp., *Ralstoniapickettii*, *Rhodanobacter spathiphylli*, *Rhodanobacter* sp., *Pseudomonasthivervalensis* and *Chryseobacterium gallinarum*. Bacterial densities in the samples ranged from 8 × 10^7^ to 1.2 × 10^9^ colony forming units per gram of substrate.

**Conclusions:**

The study shows the isolation of some potential plant and human bacterial pathogens. However, most of the isolated species were either biocontrol species or saprophytes. The study questions the ways by which these bacterial species were introduced into potting media. To the best of our knowledge, this appears to be the first report of most of the isolated bacteria from potting media, except *B. pumilus.*

## Background

Soil in arid areas of the world are known be poor in fertility and structure. This motivates growers to use various biological and chemical amendments to improve soil in their fields. Potting medium is a growing medium suitable for the establishment and development of a wide range of plants in containers. Several types of potting media are imported from European countries (Al-Sadi et al. 2011). They are mainly used for growing potted plants such as citrus, mango and ornamental plants as well as for the germination of several vegetable crops before transplanting into fields.

Studies provided evidence that potting media may harbor some plant pathogenic fungi (Al-Sadi et al. 2011; Al-Sa’di et al. 2008). In addition, potting media can also be an important source of several beneficial fungal species that can be used as bicontrol agents or as decomposers of plant residue material (Al-Sadi et al. 2015; Al-Mazroui and Al-Sadi 2015). However, limited studies addressed the occurrence of bacterial communities in potting media (Whiley et al. 2011; Lindsay et al. 2012).

Using molecular technologies, the studies of microbial ecology have been made easy (Querido et al. 2013). Studies have shown that sequence analysis of the 16S rRNA gene is an important molecular tool for the identification of bacterial species (Jagielski et al. 2014). This study was carried out to characterize the bacterial communities in potting media originating from seven countries. Knowledge into this area may help understand the potential occurrence of plant pathogenic bacteria and other bacterial types in these growing media.

## Results and discussion

Detection of bacteria in the potting media products revealed that all products contain at least one species of bacteria, except for product #13 (Norway) which was found to be free of culturable bacteria. Densities of bacterial colonies were generally very high, ranging from 8 × 10^7^ to 1.2 × 10^9^ colony forming units per gram of substrate. The four products from Germany were found to contain different types of culturable bacteria, with product #2 having the highest number of bacterial species, which was 4. Commercial products from the other countries contained 1–2 culturable bacterial species (Table 1).

**Table 1:** Detection of bacteria isolates in 13 commercial potting media products originating from seven countries

Product no.^a^	Origin	Composition	Isolate code^b^	Bacterial species	GenBank acc. no.^c^
1	Germany	White peat (particle size: 0–7 mm), with 95–99 % organic matter	SQU P001	*B. pumilus*	KU220846
			SQU P002	*L. lichenia*	KU220847
2	Germany	30 % white peat (particle size: 0–7 mm) and 70 % frozen black peat; fine structure	SQU P003	*B. phenazinium*	KU220848
			SQU P004, 5	*Burkholderia* sp.	KU220849
			SQU P006	*K. flava*	KU220851
			SQU P007	*B. vallismortis*	KU220852
3	Germany	High bog peat in fine structure (particle size: 0–7 mm)	SQU P008	*A. livingstonensis*	KU220853
4	Germany	20 % green compost, 40 % white peat (particle size: 0–25 mm) and 40 % frozen black peat; medium structure	SQU P009	*P.* *thivervalensis*	KU220854
5	Denmark	White and dark sphagnum peat, fine structure	SQU P010, 11	*B. vallismortis*	KU220855
6	Estonia	Black peat and white peat (particle size: 0–6 mm); at least 92 % organic matter	SQU P012	*Burkholderia* sp.	KU220857
7	Estonia	Black and white milled peat (particle size 6–20 mm); at least 95 % organic matter	SQU P013	*Burkholderia* sp.	KU220858
			SQU P014	*R.* *pickettii*	KU220859
8	Estonia	Peat moss (particle size: 1–10 mm); at least 95 % organic matter	SQU P015	*R.* *pickettii*	KU220860
9	Latvia	Sphagnumfuscum peat (particle size: 0–10 mm)	SQU P016	*C. gallinarum*	KU220861
			SQU P017	*Burkholderia* sp.	KU220862
10	Ireland	Sphagnum moss peat (particle size: 0–14 mm)	SQU P018	*Rhodanobacter* sp.	KU220863
11	Ireland	Sphagnum moss peat (particle size: 0–14 mm)	SQU P019	*S. warneri*	KU220864
12	Finland	White and dark sphagnum peat, fine structure	SQU P020	*R. spathiphylli*	KU220865
13	Norway	Container mix, 10 % vermiculate and 15 % perlite coarse	–	–	

^a^Manufacturers of the products are kept anonymous

^b^The sign (–) indicates that no bacteria were isolated from the product

^c^The 16S rRNA gene sequences were deposited in GenBank for the isolated bacteria

**Fig. 1:**
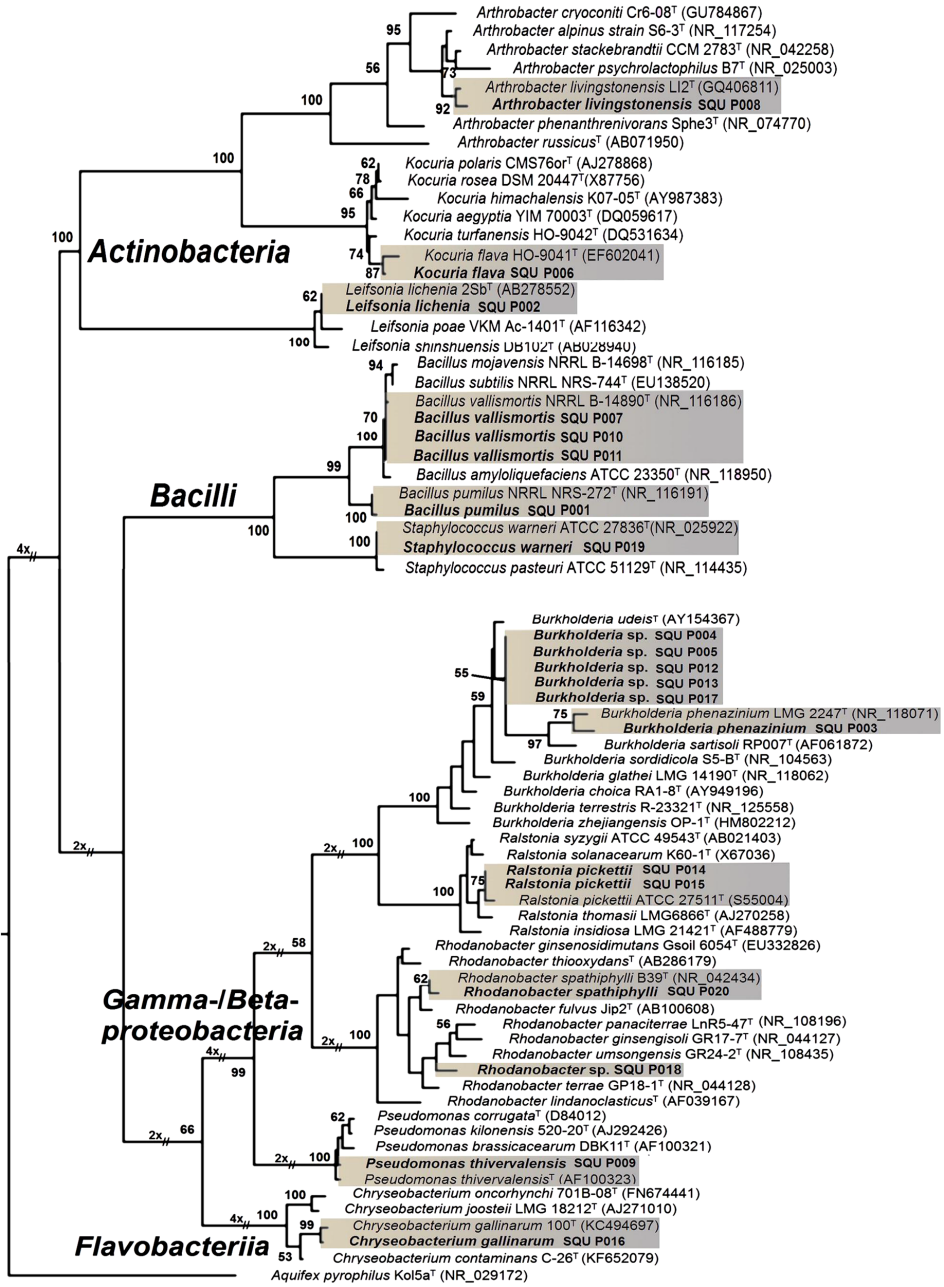
The single most parsimonious tree generated from maximum parsimony analysis of the 16S rRNA gene sequences of 20 bacterial isolates from this study (SQU P001–SQU P020) and 54 bacterial type strains obtained from the GenBank. The *bar* indicates nucleotide substitutions per site. Numbers of bootstrap support values ≥50 % based on 1000 replicates

PCR amplification of the 16S rRNA gene of all bacterial isolates produced DNA fragments ca. 1193–1228 bp long. The sequences of all isolates were deposited in GenBank (Table 1). The final sequence alignment of the dataset had 1254 characters, of which 649 characters were constant, 91 were parsimony uninformative and 514 characters were parsimony informative. MP analysis yielded a single most parsimonious tree [tree length (TL) = 1803, consistency index (CI) = 0.552, retention index (RI) = 0.923 and rescaled consistency index (RC) = 0.510] (Fig. 1).

Phylogenetic analysis revealed that the isolates were grouped in the classes *Actinobacteria*, *Bacilli*, *Gammaproteobacteria*, *Betaproteobacteria* and *Flavobacteriia*. The isolates belonging to *Betaproteobacteria* (40 %) were the most abundant, followed by *Bacilli* (25 %), *Actinobacteria* and *Gammaproteobacteria* (15 %) and *Flavobacteriia* (5 %). The isolates were identified as *Arthrobacter livingstonensis*, *Kocuriaflava*, *Leifsonia lichenia*, *Bacillus vallismortis*, *Bacillus pumilus*, *Staphylococcus warneri*, *Burkholderia phenazinium*, *Burkholderia* sp., *Ralstoniapickettii*, *Rhodanobacter spathiphylli*, *Rhodanobacter* sp., *Pseudomonasthivervalensis* and *Chryseobacterium gallinarum* (Table 1; Fig. 1).

*Bacillus* species colonies were generally opaque while *R. spathiphylli, S. warneri*, *C. gallinarum*, *L. lichenia* and *K. flava* were yellow. *B. phenazinium* was slightly beige, *A. livingstonensis* was off-white and *R.pickettii* and *P.thivervalensis* were white.

In previous studies, some bacterial species such as *Bacillus* species (Huang et al. 2012), and *Legionella longbeachae* (Koide et al. 2001), have been isolated and identified from various potting media or their ingredients such as compost, vermicompost and peat. Three bacterial species found in this study, including *B. pumilus* (Reddy 2014), *B. vallismortis* (Zhao et al. 2010) and *R. spathiphylli* (De Clercq et al. 2006) have been used as biological control agents. Moreover, *P.thivervalensis* (Achouak et al. 2000) is pathogenic to plants, and *S. warneri* (Kloos 1980) and *R. pickettii* (Stelzmueller et al. 2006) have been reported to cause diseases in humans. The other species, including *L. lichenia*, *C. gallinarum*, *A. livingstonensis*, *B. phenazinium* and *K. flava* have been previously reported from lichen (An et al. 2009), chicken (Kämpfer et al. 2014), moss-covered soil (Ganzert et al. 2011), soil (Bell and Turner 1973) and air (Zhou et al. 2008), respectively.

Differences in bacterial species composition and densities among different products could be related to differences in raw material from which the substrates were produced as well as differences in processing between different companies. For example, substrate #2, from which four bacterial species were recovered, was produced from different material (white and frozen black peat) (Table 1). The origin of bacteria in potting media products could be from the original plant waste products from which the potting media were produced (Messiha et al. 2009) or they could have been introduced during the processing or packaging of potting media (Al-Sadi et al. 2011). However, future studies might be required to investigate the source of contamination of these commercial products of potting media.

## Conclusions

Our study reveals the association of different types of bacterial species with potting media, with some being potential pathogens of plants and humans, while others are potential biocontrol species or saprophytic species. To our knowledge, this is the first report of culturable *A. livingstonensis*, *K.flava*, *L. lichenia*, *B. vallismortis*, *B. pumilus*, *S. warneri*, *B. phenazinium*, *Burkholderia* sp., *R.pickettii*, *R. spathiphylli*, *Rhodanobacter* sp., *P.thivervalensis* and *C. gallinarum* from potting media.

## Methods

### Collection of samples and isolation of bacteria

In this study, 13 samples of potting media from different European countries were obtained from Sultan Qaboos Sea Port (Table 1). Each sample represents a different company. Bacteria were isolated using serial dilution technique on nutrient agar media (NA, OXOID, England, UK). In this technique, a sample suspension was prepared by adding 1 g potting medium sample to 10 ml sterile distilled water and mixed well for 15 min and vortexed. Each suspension was serially diluted 10^−1^–10^−4^. Then 0.2 ml was pipetted from the 4th dilution onto NA media, spread with a sterile glass spreader and incubated at 28 °C for bacterial observation. Bacterial colonies which appeared different in morphology or color from each other were transferred to new NA media. Eventually, the obtained isolates were maintained on NA slant agar at 4 °C as stock culture.

### DNA extraction and PCR

For DNA extraction, single bacterial colonies were transferred to nutrient broth in 1.5 ml broth and incubated in a shaker (120 rpm) at 28 °C for 48 h. Total genomic DNA was extracted from all bacterial isolates using GenElute Bacterial Genomic DNA Kit (Sigma Aldrich, Germany) according to the manufacturer’s protocol. For PCR amplification, the 16S rRNA gene fragment was amplified using the universal primers 518F (5′-CCAGCAGCCGCGGTAATACG-3′) and 800R (5′-TACCAGGGTATCTAATCC-3′) using PuRe Taq Ready-To-Go PCR beads (GE Healthcare UK Limited, UK). Thermocycling was carried out with the following conditions: heating at 95 °C (2 min); then 40 cycles of 95 °C (40 s), 60 °C (1 min) and 72 °C (1 min); and a final extension step at 72 °C (10 min). The PCR products were sequenced using a commercial sequencing service provider (Macrogen Inc., Seoul, Korea).

### Phylogenetic analysis

Sequences of each isolate were refined using BioEdit sequence Alignment Editor (Hall 1999), in which the sequences obtained from reverse primers were transformed to the reverse complement orientation and aligned with the sequences obtained from forward primers to obtain consensus sequences. To analyze the relationships of the isolates to known bacterial species, the 20 sequences from this study, 53 sequences of type strains which had the closest relationship to the isolates and *Aquifex pyrophilus* (type strain Kol5a) as an outgroup were initially aligned using the Clustal W Multiple alignment (Thompson et al. 1994), checked visually and improved manually where necessary. Phylogenetic analysis of the 16S rRNA gene using the parsimony optimality criterion was performed in PAUP* 4.0b10 (Swofford 1998). Gaps were treated as missing data. Maximum parsimony (MP) analysis was conducted by heuristic searches consisting of 1000 stepwise random addition replicates and branch swapping by the tree-bisection-reconnection algorithm. For each MP analysis, 1000 bootstrap replicates using a heuristic search with simple sequence addition was performed to assess statistical support for branch stability.
